# Filling the Gap in Southern Europe—Diversity of *Cryphonectria parasitica* and Associated Mycovirus (*Cryphonectria hypovirus 1*) in Montenegro

**DOI:** 10.3390/jof8060552

**Published:** 2022-05-24

**Authors:** Lucija Nuskern, Milena Stojanović, Marija Milanović-Litre, Tena Šibenik, Marin Ježić, Igor Poljak, Mirna Ćurković-Perica

**Affiliations:** 1 Division of Microbiology, Department of Biology, Faculty of Science, University of Zagreb, Marulićev trg 9a, 10000 Zagreb, Croatia; lucija.nuskern@biol.pmf.hr (L.N.); mlitremarija@gmail.com (M.M.-L.); tena.sibenik@gmail.com (T.Š.); marin.jezic@biol.pmf.hr (M.J.); 2 Biotechnical Faculty, University of Montenegro, Mihaila Lalića Br. 15, 81000 Podgorica, Montenegro; milenas@ucg.ac.me; 3 Department of Forest Genetics, Dendrology and Botany, Faculty of Forestry and Wood Technology, University of Zagreb, Svetošimunska Cesta 23, 10000 Zagreb, Croatia; ipoljak@sumfak.hr

**Keywords:** chestnut blight, *Cryphonectria hypovirus 1* (CHV1), biocontrol, invasive pathogens, sweet chestnut

## Abstract

*Cryphonectria parasitica* is an invasive fungal pathogen that causes blight disease on chestnut trees. Its destructive effect can be controlled with naturally occurring mycovirus *Cryphonectria hypovirus 1* (CHV1). To date, the spread of *C. parasitica* and CHV1 in Europe is fairly well documented, but there are still several unexplored regions. Thus, we sampled blight cankers from four sweet chestnut populations in Bay of Kotor and Lake Skadar regions in Montenegro. We determined vegetative compatibility (vc) type and mating-type diversity using molecular *vic* and MAT1 genotyping, as well as confirming the presence of CHV1 by RT-PCR. We identified 11 vc types, with EU-12 being the dominant one represented by 58.2% of all fungal isolates. The Shannon diversity index ranged from 0.93 to 1.47. Both mating types of *C. parasitica* were found in all four populations. The prevalence of CHV1 ranged from 15% to 40%. All CHV1 isolates belonged to Italian subtype I of CHV1 and were closely related, with relatively recent common ancestors. Our results indicate a longer presence of *C. parasitica* and CHV1 in Montenegro than previously thought. Natural biocontrol with CHV1 seems to be well established. However, it has the potential for deterioration; thus, close monitoring is required.

## 1. Introduction

Invasive alien pathogens and pests (IAPP) pose a global threat to biodiversity, but also lead to the vast economic losses due to their impact on agriculture and forestry. Among IAPP organisms, plant pathogenic fungi play an important role, causing greater losses than non-indigenous insects, and are able to transform forest and rural landscapes in just several decades [1,2]. One of the unfortunate examples of such destructive power is the ascomycete fungus *Cryphonectria parasitica*, also called the chestnut blight fungus, listed as one of the top 100 most dangerous invasive species according to the Global Invasive Species Database [3]. It primarily inhabits the bark of chestnut trees (*Castanea* spp.), and causes chestnut blight disease on susceptible species [4]. Aside from chestnuts, *C. paracitica* has been sporadically observed on other tree genera such as *Quercus* L., *Acer* L., *Alnus* (L.) Gaertn., *Eucalyptus* L’Hér., and *Ostrya* Scop., although the disease symptoms are mild and the infection is not lethal [5]. Artificial inoculations on *Quercus robur* L. and *Fagus sylvatica* L. [6] also suggest its weak pathogenicity on other common European tree species. Since its introduction to the USA from Asia in 1904, it has nearly eliminated American chestnut (*C. dentata* (Marshall) Borkh.), formerly the dominant tree species [7]. In Europe, *C. parasitica* was first detected on European sweet chestnut (*C*. *sativa* Mill.) in 1938 in Italy near Genoa, from where it spread, causing substantial damage [7]. The spread of invasive alien pathogens is often rapid and deleterious due to the higher host susceptibility in the new non-native range, arising from the absence of coevolution with the indigenous hosts [2]. General lack of knowledge about the distribution, host ranges, and pathogenicity of most fungal species [8], uncertain prediction of disease outbreaks, and high costs of control measures [1] make the surveillance of the spread of alien fungi of primary concern. In parallel, the focus is on finding self-sustainable long-term measures to control the pathogen populations, and biocontrol is considered a promising method in this regard [9].

Despite the vast damage it caused in the USA, in Europe, after an aggressive start of the epidemic, *C. parasitica* was soon discovered to harbor naturally present biocontrol agent—an unencapsidated RNA mycovirus named *Cryphonectria hypovirus 1* (CHV1). This virus causes hypovirulence—a persistent infection of the fungus that reduces its virulence, sexual reproduction, pigmentation, and asexual sporulation [10]. Hypovirulent *C. parasitica* does not cause the aggressive form of the blight disease; rather, it stays localized on the surface of the bark and has a protective effect, as it is the source of CHV1 infective inoculum for other uninfected, virulent fungi. On the other hand, when an aggressive bark canker, already infected with virulent form of *C. parasitica*, secondarily acquires CHV1, the growth of the mycelium is slowed down, thus enabling the tree to form a callus and heal the wound [11].

The spread of *C. parasitica* in a new region and the success of biocontrol depend on several parameters of fungal population. Namely, CHV1 spreads between fungal individuals by hyphal anastomosis, which in fungi is limited by self–non-self-recognition system called vegetative compatibility (vc) [12]. In European *C. parasitica* populations vc is controlled by at least six diallelic *vic* loci defining 64 different vc types, also known as EU-types [13]. Additional vc types have been detected; however, their genetic background has not been determined yet [14]. The hypovirus will easily spread between compatible fungal individuals, i.e., the ones with the same allelic composition on all *vic* loci. In case of contact between incompatible hyphae, the anastomosis is not stable and the programmed cell death occurs, hindering the spread of the virus [13]. Consequently, in *C. parasitica* populations with low vc type diversity, CHV1 is easily disseminated in the population, while high vc-type diversity complicates the process [14,15]. The vc-type diversity of *C. parasitica* in a certain population can increase either by introduction of new vc types, i.e., with infected plant material or by natural spore dispersal, or by sexual reproduction between individuals of opposite mating types inside a population [4]. Sexual reproduction hinders the spread of the hypovirus in two ways: firstly, it increases the vc-type diversity because new vc types can arise through recombination of polymorphic *vic* genes [16], and secondly, unlike the asexual spores, sexually derived ascospores do not transmit the virus [17]. Thus, knowledge of the occurrence and diversity of *C. parasitica* vc types, as well as the information on presence and ratio of both mating types in a certain population, is essential for the assessment of potential for natural biological control.

To date in Europe, *C. parasitica*, but luckily the accompanying biocontrol agent as well, has been detected throughout the sweet chestnut growing regions, from Portugal in the west [18] to Romania and Ukraine in the east [19], and from Germany in the north [20] to Greece in the south [21]. Throughout its range in Europe, the diversity of *C. parasitica* is highly variable, although generally its genetic diversity is far lower than in its native range in eastern Asia, where it coevolved with Asian chestnut species *C*. *crenata* Siebold et Zucc., *C. mollissima* Blume, *C. henryi* (Skan) Rehder et E. H. Wilson and *C. seguinii* Dode [22,23]. In northwestern and central Europe, where *C. parasitica* was first introduced and established, the populations of *C. parasitica* are more diverse than the populations in the southeast at the expanding front of the chestnut blight epidemic [24]. Although the spread of *C. parasitica* in Europe is fairly well-documented, there are still some unexplored regions, such as Montenegro. In this country, as well as in the rest of the Balkan Peninsula, sweet chestnut is a species with high economic importance, since it is used for timber of excellent technical value as well as for food, honey production, and as a medicinal herb [25]. Chestnut blight was first documented in Montenegro some 50 years ago near Ostros (Lake Skadar), yet although some fungal samples have been isolated and morphologically characterized as orange (i.e., virulent) or white (i.e., hypovirulent) [26], no further population studies regarding the diversity of the fungus nor hypovirus have been conducted.

Thus, in this study we: (1) determined vc and mating types of *C. parasitica* isolates sampled from four populations in Montenegro, (2) estimated vc type diversity and assessed the potential for an increase in that diversity, and (3) determined the prevalence and genetic diversity of CHV1 present in sampled populations in order to fill the gap in our knowledge about the spread of this dangerous pathogen and the associated biocontrol agent.

## 2. Materials and Methods

### 2.1. Sampling

**Figure 1:**
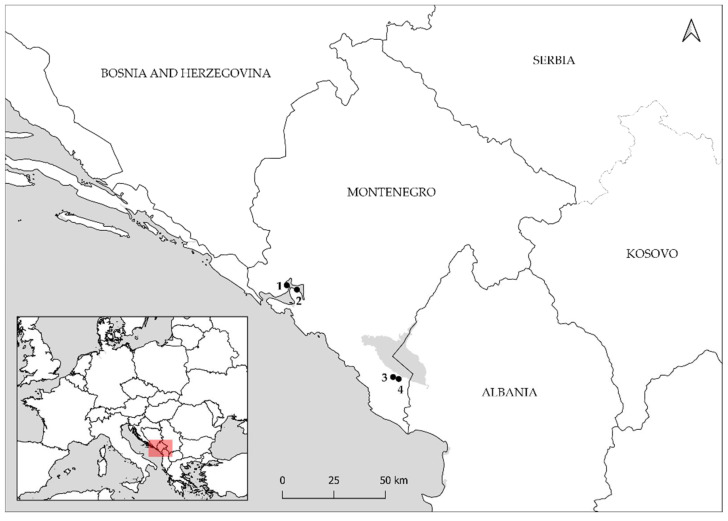
Locations of four sampled *Cryphonectria parasitica* populations in Montenegro: 1 = Bay of Kotor–Kostanjica (BK-K), 2 = Bay of Kotor–Stoliv (BK-S), 3 = Lake Skadar–Koštanjica (LS-K), 4 = Lake Skadar–Ostros (LS-O). Red rectangle on the map of Europe shows the area of interest.

The *Cryphonectria parasitica* samples were collected in late autumn of 2019. Due to the poor success of *C. parasitica* isolation, the cankers from which no colonies of the fungus were obtained were resampled in mid spring of 2020. The sampling was performed in four chestnut-growing areas in Montenegro: two at Lake Skadar–Koštanjica (LS-K) and Ostros (LS-O), and two at Bay of Kotor–Stoliv (BK-S) and Kostanjica (BK-K) (Figure 1). Lake Skadar populations grow on elevations of up to 250 m above sea level, in a sub-Mediterranean climate zone in acidic brown soil over the limestone bedrock [25,27]. The type of climate in Bay of Kotor is Mediterranean and chestnut populations are situated at low elevations (from about 20 m up to 200 m above sea level), on steep terrain with acidic soil [27,28]. Chestnut trees with cankers were randomly chosen and only one canker per tree was sampled. Twenty-one cankers were sampled at LS-K, 21 at LS-O, 31 at BK-S and 30 in BK-K. From each canker, three bark plugs were collected with a 2 mm bone marrow biopsy needle: one near the upper margin, one in the center, and one near the lower margin of the canker. Between each sampling, the needle was sterilized by dipping in 96% ethanol and flaming. The cankers were characterized according to Ježić et al. [29] as: active (aggressive form of the disease with canker expanding in the bark and the cambium), callus (healing of the canker by formation of callusing tissue around the wound), necrosis (mild form of the disease with slowly growing superficial canker) or any combination of the previous for the cankers with combined morphology between these three categories. The canker assessment was always performed by the same person to minimize the discrepancies between samplings.

### 2.2. Sample Cultivation

The bark plugs were surface-sterilized in 70% ethanol for several seconds, then dried on sterile filter paper. Three bark plugs from the same canker were placed on Petri plates (∅ 90 mm) containing approximately 20 mL of potato dextrose agar (PDA; Difco, BD, Franklin Lakes, NJ, USA) and incubated in a growth chamber (Pol-eko aparatura, Wodzisław Śląski, Poland) at 24 °C and 70% humidity in the dark. After several days, hyphal tips from the freshly grown mycelia expanding from bark samples were transferred to new Petri plates (∅ 60 mm) with fresh PDA. To obtain as many isolates as possible, visibly contaminated samples were then transferred to PDA supplemented with ampicillin (Roth, Karlsruhe, Germany) at 200 mg/L and rifampicin (Roth, Karlsruhe, Germany) at 10 mg/L and grown for several days, after which hyphal tips were subcultured on fresh PDA. The obtained single cultures of *C. parasitica* were subsequently incubated in a growth chamber at 24 °C and 70% humidity in the dark for seven days and in light for seven days to enable the sporulation. The obtained cultures were then stored in 22% glycerol at −80 °C until needed for the experiments.

### 2.3. Isolation of Nucleic Acids

For isolation of nucleic acids, one cube of glycerol stock culture was placed on a ∅ 60 mm Petri plate containing 10 mL of potato dextrose agar overlaid with cellophane. The cultures were incubated for 10 days in climate chamber in the dark at 24 °C and 70% relative humidity. After 10 days mycelia were stripped from the cellophane, transferred to the 2 mL Eppendorf tube, and lyophilized for 24 h. Lyophilized tissue was ground to a fine powder with a ∅ 5 mm steel ball in TissueLyser II (Qiagen, Venlo, The Netherlands) for 2 min at 30 Hz.

Total genomic DNA was extracted from 9–11 mg lyophilized tissue with OmniPrep^TM^ for Fungus (G-Biosciences, St. Louis, MO, USA) according to the manufacturer’s instructions. The final DNA extract was dissolved in PCR-quality water. Total RNA was extracted from 3–5 mg lyophilized tissue with a GenElute™ Total RNA Purification Kit (Sigma-Aldrich, St. Louis, MO, USA). Concentrations of DNA and RNA were measured with NanoDrop 2000c (ThermoFisher Scientific, Waltham, MA, USA). The samples were stored at −20 °C for DNA and −80 °C for RNA, until they were used in further experiments.

### 2.4. Molecular vic Genotyping

Molecular *vic* genotyping was performed by multilocus PCR assay according to the methods of Short et al. and Mlinarec et al. [30,31]. Amplification of specific *vic* regions was carried out in total of 10 µL according to Mlinarec et al. [31] using GoTaq G2 Flexi DNA Polymerase (Promega, Madison, WI, USA), as in all other PCR reactions. We used primer sets vic1a, vic2, vic3a, vic6, and vic7 from Short et al. [30] and vic4 from Mlinarec et al. [31]. PCR products were analyzed in 1% agarose gel pre-stained with GelStar nucleic acid dye (Lonza, Basel, Switzerland), prepared and run in 0.5× AccuGENE TBE (Lonza, Basel, Switzerland) and run at 5 V/cm for 60 min.

### 2.5. Mating Type Determination

The mating type of all isolated samples was determined by PCR using primer pair M1-GS1 and M1-GS2-rev to amplify the MAT1-1 idiomorph and primer pair M2-GS2 and InvA5n to amplify the MAT1-2 idiomorph [32]. Amplification was carried out in a total of 15 µL with concentrations and cycling conditions, as in Krstin et al. [33]. PCR products were analyzed in 0.7% agarose gel prepared as described previously and run at 5 V/cm for 105 min.

### 2.6. CHV1 Detection

All samples were molecularly analyzed with RT-PCR. First-strand cDNA synthesis was performed with High-Capacity cDNA Reverse Transcription Kit (Applied Biosystems™, Waltham, MA, USA) according to the manufacturer’s directions. Samples with RNA concentration higher than 200 ng/µL were first diluted to that concentration. For ORF-A amplification, primer pair hvep1 and EP721-4 [34,35] was used, and for ORF-B amplification, primer pair EP713-6 and EP713-7 was used [36]. In all steps, appropriate positive, negative, and water controls were used. For ORF-A, touchdown PCR with the following conditions was applied: initial denaturation step for 2 min at 94 °C followed by 10 cycles consisting of denaturation for 1 min at 94 °C, annealing for 1:30 min at 60 °C, and extension for 2 min at 72 °C, with the annealing temperature reduced 0.5 °C per cycle. After that followed 25 cycles of denaturation for 1 min at 94 °C, annealing for 1:30 min at 55 °C, and extension for 2 min at 72 °C with the final extension for 10 min at 72 °C. For ORF-B, the following cycling conditions were applied: 2 min at 94 °C followed by 35 cycles of denaturation for 1 min at 94 °C, annealing for 1:30 min at 55 °C, and extension for 2 min at 72 °C, with the final extension for 10 min at 72 °C. PCR products were visualized in 1% agarose gel prepared as described previously and run at 5 V/cm for 60 min. After confirming the presence of desired fragments, PCR amplicons were sent for sequencing to Macrogen Europe B.V. (Amsterdam, The Netherlands).

### 2.7. Data Analysis

The maps were created using the free and open source QGIS (version 3.10 A Coruña; QGIS.org, 2022. QGIS Geographic Information System. QGIS Association). National borders and water surface data were derived from © OpenStreetMap contributors (https://www.openstreetmap.org/copyright (accessed on 8 March 2022)) and Eurostat—GISCO (https://ec.europa.eu/eurostat/web/gisco/ (accessed on 8 March 2022)).

The diversity of vc types in each population was assessed by the number of vc types (*S*), Shannon diversity index (*H*′) and Buzas and Gibson’s evenness (*E*, where $E=\frac{e^{H’}}{S}$). The *H*′ and *E* were calculated in Past3 [37]. For mating types present in each population, the deviation from 1:1 ratio was tested with the χ-squared test, calculated in Microsoft Excel. The differences in frequencies median between virulent and hypovirulent isolates belonging to different vegetative compatibility types were tested with Wilcoxon test in Past3.

### 2.8. Sequence Analysis

All raw sequencing data were screened manually in GeneStudio Pro 2.2 (GeneStudio, Inc., Suwanee, GA, USA) to ensure that the bases were scored correctly. The assembly of nucleotide sequences was also performed in GeneStudio Pro 2.2 with CHV1 strain EP721 (accession number DQ861913) as reference sequence. Sequence alignment was performed in MEGA 7.0.21 [38] using ClustalW and default parameters for alignment. Sequences of ORF-A were deposited in GenBank under the accession numbers ON180782–ON180802, and ON180803–ON180823 for ORF-B. For subsequent analyses, the sequences were trimmed to the length of the shortest sequence. We calculated the number of nucleotide differences among CHV1 sequences (k), the number of variable sites, nucleotide diversity (π), the number of indel sites and haplotypes, and the total number of haplotypes and haplotype diversity (Hd) using DnaSP v6 [39].

The evolutionary history between CHV1 isolates from Montenegro was inferred using the Maximum Likelihood (ML) method based on the Tamura-Nei model [40]. The bootstrap consensus tree inferred from 1000 replicates was taken to represent the evolutionary history of the taxa analyzed. For analysis of both ORF-A and ORF-B sequences obtained from Montenegro CHV1 strain EP713 (accession number NC_001492) was used as the root of the tree. Standard strains EP721 and Euro7 (accession numbers DQ861913 and AF082191, respectively) were also used in analysis. To place the Montenegrin CHV1 sequences in context of the surrounding countries, haplotype network was constructed in PopArt (http://popart.otago.ac.nz (accessed on 15 February 2022)) with a minimum spanning network model [41] using partial ORF-A sequences from Croatia, Bosnia and Herzegovina and North Macedonia obtained from GenBank (NCBI) (https://www.ncbi.nlm.nih.gov/nucleotide/ (accessed on 9 February 2022)). All the samples and their accession numbers are listed in Table 1.

## 3. Results

### 3.1. Diversity of Cryphonectria parasitica Vegetative Compatibility Types

Out of 103 cankers sampled across four populations in Montenegro, we isolated 79 *C. parasitica* samples from 60 cankers (Table 2). Five cankers in BK-K, three in BK-S, one in LS-K and one in LS-O yielded multiple *C. parasitica* isolates, and in some cases, those multiple isolates were of discordant vc types. A total of 11 vc types was identified across sampled populations, with EU-12 being the dominant one (58.2%). The number of vc types identified in each population ranged from four, found in Bay of Kotor-Kostanjica, to six, in Lake Skadar-Ostros. Interestingly, despite having the largest number of vc types present in the population, due to the high isolation efficacy and subsequent collection of rare types, Ostros was clearly dominated by a single vc type—EU-12 (72.0%). In other populations, EU-12 was also the most abundant vc type, but in populations BK-S and LS-K, it was co-dominated by EU-2 (39.1%) and EU-5 (27.3%), respectively. In BK-K, the most abundant vc type after EU-12 (65%) was EU-2 (25.0%).

The diversity of vc types calculated with the Shannon diversity index (*H*′) ranged from 0.93 in BK-K to 1.47 in LS-K (Table 3). The evenness of populations in Montenegro varied from 0.46 in LS-O, dominated by EU-12, to 0.87 in LS-K, where the three most common vc types, EU-12, EU-5, and EU-6, occurred at frequencies of 36.4, 27.3, and 18.2%, respectively.

Based on known *vic* genotypes, number of polymorphic *vic* loci was determined, and subsequently, the maximum theoretic number of vc types was calculated for each population (Table 3). Both populations from Bay of Kotor had only three polymorphic *vic* loci, leading to eight possible vc types, while in the population LS-O, all six *vic* loci were polymorphic, which could potentially give rise to all 64 known European vc types.

### 3.2. Diversity of Cryphonectria parasitica Mating Types

Both mating types of *C. parasitica* were found in all four of the studied chestnut populations from Montenegro, with MAT1-1 being the dominant one across all samples (75.9%). Mating-type ratios were tested for deviation from 1:1 for each population using the χ-squared test (Table 3). The mating type ratio was significantly different from 1:1 in populations BK-S and LS-O, but not in BK-K and LS-K. In populations from Bay of Kotor (BK-K and BK-S), both mating types were found in the dominant vc type EU-12, as well as in the second most abundant vc type, EU-2. On the other hand, in populations from Lake Skadar (LS-K and LS-O) in the dominant EU-12 vc type, only MAT1-1 was present.

### 3.3. CHV1 Prevalence and Diversity

The presence of hypovirus CHV1 was detected in all sampled populations from Montenegro, with total prevalence of 26.6% in fungal isolates and 23.3% in cankers (Table 4). Due to multiple fungal isolates from the same canker (BK-K_16-1 and 16-2; BK-S_07-1, 07-2 and 07-3; LS-O_05-1 and 05-3; 06-1 and 06-2; 09-2 and 09-3; 14-1 and 14-2), in some populations, the number of CHV1-positive isolates was higher than the number of hypovirulent cankers (i.e., cankers with CHV1-positive *C. parasitica* isolates). The CHV1 prevalence in fungal isolates ranged from 15.0% in population BK-K to 40.0% in LS-O. When focusing only on hypovirulent cankers (ignoring multiple isolates from the same canker), the average prevalence in Bay of Kotor populations (BK-K and BK-S) was 15.0%, while in Lake Skadar populations (LS-K and LS-O), it was 32.6%. CHV1 was detected in the most frequent vc type EU-12 in all populations except LS-K. It was also detected in the second most frequent vc type EU-2 in BK-S, as well as in EU-5 and EU-6 in LS-K. The Wilcoxon test showed that there was no statistical difference in frequencies of CHV1 presence between different vc types (z = 0.62392; *p* = 0.53268) across all populations.

Across all sampled populations, a total of 21 CHV1 isolates were identified and partially sequenced in ORF-A and ORF-B regions. For the sequenced region of ORF-A, the length of the obtained sequences was 609 nt, spanning positions 1530–2138 in the reference genome. We detected a total of 34 variable sites, with nucleotide diversity π= 0.01136 ± 0.00085 and the average number of nucleotide differences per site k = 6.90476. Among the detected mutations, one was a one-nucleotide deletion observed in two haplotypes (BK-S_07-2, BK-S_29-3) and the rest were nucleotide substitutions. A total of 17 different haplotypes were detected with haplotype diversity Hd = 0.981 ± 0.020. For the ORF-B region, the length of the obtained sequences was 1199 nt, spanning positions 8823–10,021 in the reference genome. The number of variable sites was 75, with nucleotide diversity π= 0.01240 ± 0.00087, and the average number of nucleotide differences per site k = 14.86190. Nucleotide substitutions were the only type of mutations observed in this partial ORF-B sequence. A total of 18 different haplotypes were detected with haplotype diversity Hd = 0.986 ± 0.019.

**Figure 2:**
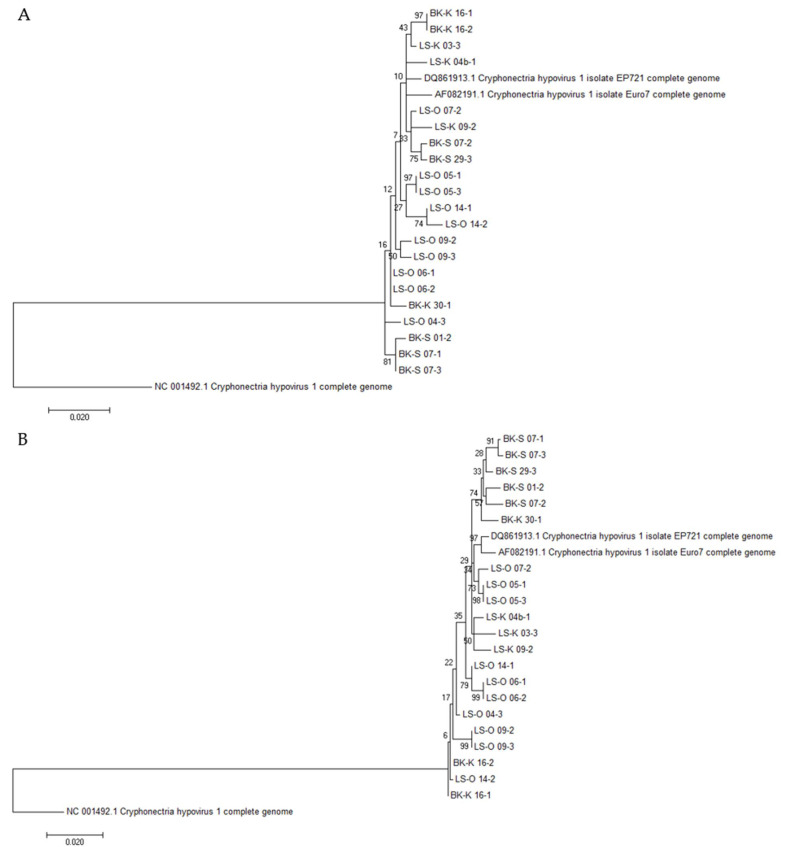
Phylogenetic tree of *Cryphonectria hypovirus 1* (CHV1) sequences from Montenegro constructed with maximum likelihood method. Figure in (**A**) is inferred from 609 nt long part of ORF-A, and in (**B**) from 1199 nt long part of ORF-B. Reference CHV1 sequences Euro7 (accession number AF082191) and EP721 (accession number DQ861913) were also included in the analysis, and EP713 (accession number NC_001492) was used as the root of the tree. All reference sequences have been truncated to the same region as analyzed sequences. Bootstrap values of 1000 replicates are indicated on branches.

Phylogenetic analysis of partially sequenced ORF-A and ORF-B regions from Montenegro clustered all CHV1 isolates together with reference sequences Euro7 and EP721, placing them within the Italian subtype I (Figure 2). The topologies of the trees for ORF-A and ORF-B differed. In the ORF-A region, there was no grouping of the samples according to population, but in the ORF-B region, the separation between samples from Bay of Kotor and the ones from Lake Skadar can be seen to some extent, albeit with low bootstrap support. The CHV1 isolates that came from the same canker were mostly identical or clustered together, except LS-O 14-1 and 14-2 in ORF-B region and BK-S 07-1, 07-2 and 07-3, where isolate 07-2 separated from the other two in both the ORF-A and ORF-B region.

**Figure 3:**
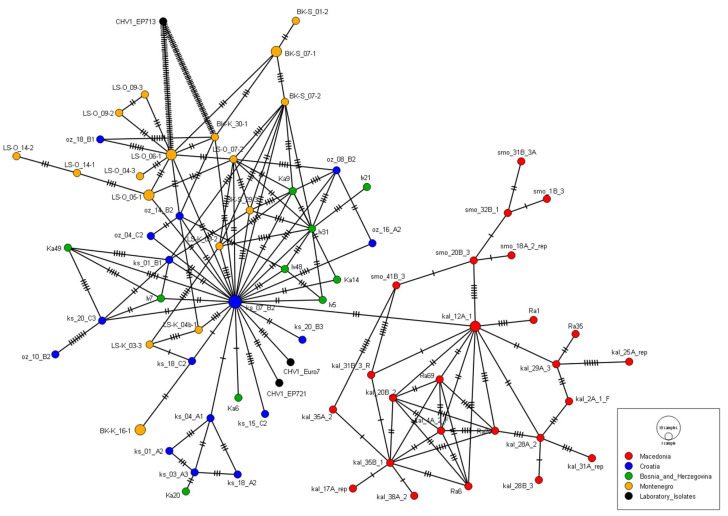
Haplotype network with 561 nt long *Cryphonectria hypovirus 1* sequences from the ORF-A region of the genome. Circle sizes correspond to the number of isolates sharing the same consensus sequence. Isolates from Montenegro are represented with orange, from Croatia with blue, from Bosnia and Herzegovina with green and from North Macedonia with red. Reference laboratory strains EP713, EP721 and Euro7 are represented with black. The bars indicate the number of single-nucleotide mutations separating two sequences.

When analyzing partial ORF-A sequences obtained from Montenegro together with sequences from North Macedonia, Bosnia and Herzegovina and Croatia retrieved from GenBank, the sequences were trimmed to the length of 561 nt, spanning position 1526–2086 in the reference genome. Haplotype network (Figure 3) separated North Macedonian sequences in a distinctive cluster, while Croatian and Bosnian sequences were grouped together, forming the central part of the network. The majority of Montenegrin sequences were grouped together and were placed somewhat closer to the reference sequence EP713 than the sequences from Croatia and Bosnia and Herzegovina.

## 4. Discussion

**Figure 4:**
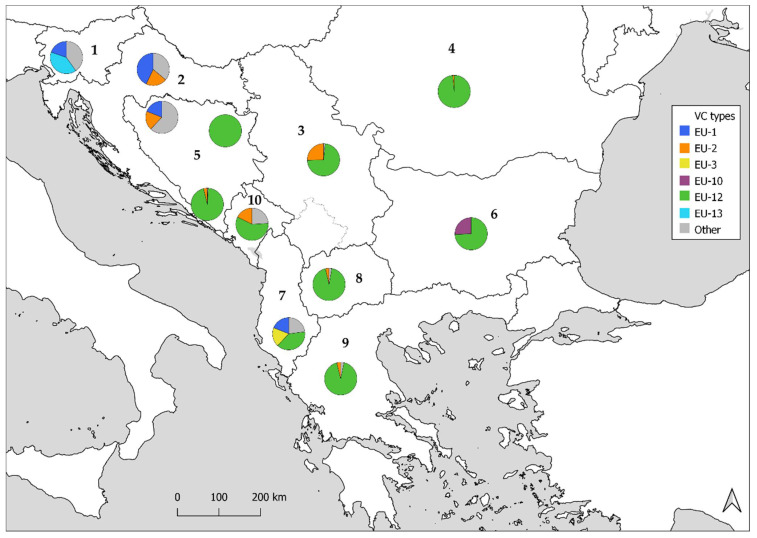
Two most abundant vegetative compatibility (vc) types of *Cryphonectria parasitica* in different countries on Balkan Peninsula, based on previously published data for Slovenia (1) [33], Croatia (2) [44], Serbia (3) [42], Romania (4) [19,47,48], Bosnia and Herzegovina (5) [45], Bulgaria (6) [47], Albania (7) [43], North Macedonia (8) [46], and Greece (9) [21,46], and data from this research for Montenegro (10).

The overall diversity of *Cryphonectria parasitica* populations in Montenegro is fairly high, as seen from the range of the Shannon diversity index from 0.93 to 1.47, despite the dominance of only one vc type—EU-12. The second most abundant vc type was EU-2, while the others were represented with less than 10% of samples. In the neighboring countries, as summarized in Figure 4, EU-12 was also the dominant vc type in Serbia [42] and Albania [43], while in Bosnia and Herzegovina it was dominant in the eastern and south-western parts of the country. In the northern part of BiH and continental Croatia, the most common vc types are EU-1 and/or EU-2 [24,29,44,45], and further to the north, in Slovenia, the dominant vc type is EU-13, followed by EU-1 [33]. In countries south of Montenegro, the dominance of EU-12 is even more pronounced at the margins of the expanding range of the pathogen: it comprises 94% of the sampled isolates in North Macedonia [46] and, depending on the sampled populations, 88–100% in Greece [21,46,47].

The observed range of Shannon diversity in Montenegro is higher than in neighboring Serbia (0.648) [42], the eastern and south-western Bosnia and Herzegovina (0 and 0.22, respectively) [45], and nearby North Macedonia (0–0.789) [46], and in a similar range as in Albania (0.693–1.565) [43] and Croatia (0.63–1.69) [44]. In Slovenia, the diversity of *C. parasitica* populations is slightly higher, ranging from 1.16 to 1.89 [33], while in north-western Bosnia and Herzegovina it was 2.52, most probably due to the depth of sampling [45]. Higher vc type diversity is generally found in regions where *C. parasitica* is present for a longer period of time, such as northern Italy (0.82–1.76), southwestern France (1.11–2.05), and southern Switzerland (1.27–2.18) [14,49]. At the invasive front of the pathogen, e.g., southern Italy [49] and Sicily [47], northern Switzerland [50], Portugal [18], Spain [51], Bulgaria, Romania, North Macedonia, and Greece [47], the diversity is much lower, and the population is usually dominated by the single vc type. Thus, although not characterized up to now, we can conclude that *C. parasitica* populations in Montenegro are most probably older than previously thought. The data on the first record of chestnut blight in Montenegro reported by Dubak [26] put Montenegrin populations in age close to the North Macedonian ones (1974). However, higher vc type diversity of *C. parasitica* populations in Montenegro found in this research suggest they might have been established at a similar time as the Croatian and Bosnian ones, where the disease was first reported in 1955 and 1961, respectively.

When comparing populations from Montenegro, a differentiation between Bay of Kotor and Lake Skadar populations can be seen. Lake Skadar populations were slightly more diverse than Bay of Kotor populations. Additionally, there was a clear distinction in observed vc types. While EU-12 was the dominant vc type in all studied populations, the only other vc type that was present in both regions of Montenegro was EU-17 (BK-S and LS-O), while all other vc types were present either only in Bay of Kotor (EU-2, EU-4, EU-8, EU-22) or the Lake Skadar region (EU-1, EU-5, EU-6, EU-10, EU-11). This could indicate a possibility of two separate introduction events of the pathogen in the territory of Montenegro. Interestingly, the population of Lake Skadar-Koštanjica, from which the fewest samples were obtained, was also the most diverse one. The 1:1 mating-type ratio in this population suggests the possibility of sexual reproduction, and could explain the observed high diversity of this population [52]. The populations also differed in the number of polymorphic *vic* loci. In both populations from Bay of Kotor we recorded the polymorphism on three *vic* loci (*vic1*, *vic6* and *vic7*), leading to a maximum of eight potential vc types. For instance, in Bay of Kotor-Kostanjica the cross between two dominant vc types, EU-12 and EU-2, could generate vc types EU-4 and EU-8 already present in the population, but also potential new types EU-14, EU-17, EU-22 and EU-29 [13]. Lake Skadar populations had higher genotypic diversity with five polymorphic *vic* loci in Koštanjica (all except *vic3*) and all six in Ostros, which could potentially give rise to 32 or all 64 vc types, respectively. In some Croatian *C. parasitica* populations, a substantial increase in vc type diversity has been detected in as little as ten years [24,29,44], and a similar increase can be expected in Montenegro, given that both mating types were found in all populations, and especially in Bay of Kotor-Kostanjica and Lake Skadar-Koštanjica, where mating type ratio suggests that sexual reproduction is most likely occurring in these populations. On the other hand, the absence of MAT1-2 allele in the dominant EU-12 vc type from Lake Skadar populations could slow down the process.

Although white *C. parasitica* isolates in Montenegro were reported previously [26], indicating CHV1 presence, here we confirmed its presence with molecular methods in all studied populations. The CHV1 prevalence ranged from 15.0% to 40.0%, which is slightly lower than found in Slovenia (11.1–72.2%) and Croatia (12.7–66.6%) [33,44], but substantially higher than in neighboring Albania (less than 1%) or Serbia, where CHV1 was not detected among 77 analyzed samples, although only culture morphology was used in the assessment [42,43]. In Europe, high CHV1 prevalence is often found in regions where chestnut blight is present for a longer period of time [24,52] and with low vc-type diversity [24,53], while low prevalence is associated with the recent introduction of CHV1 into *C. parasitica* populations [18,51,54]. In Montenegro, the prevalence of hypovirulent isolates observed suggests the long presence of the virus and its host.

Despite the higher vc-type diversity, the populations from Lake Skadar had higher CHV1 prevalence than the ones from Bay of Kotor, indicating successful spread of natural hypovirulence. It was shown previously that the level of vc type diversity in many regions in Europe does not pose a major barrier for the hypovirus spread [11]. Furthermore, the spread of natural hypovirulence in Montenegro could be facilitated by the presence of CHV1 in the dominant EU-12 vc type in all populations except Lake Skadar-Koštanjica. The high number and diversity of vc types in this population, as well as the presence of both mating types and the absence of CHV1 in the most common vc-type, make this population vulnerable to destructive spread of chestnut blight, and close monitoring in the future is advisable.

As expected, all 21 CHV1 isolates detected in this research belonged to the Italian subtype I of CHV1 [34]. Subtype I is the most widespread CHV1 subtype in Europe, and the only one found in surrounding countries [36,53,55]. The haplotype diversity of ORF-A sequences observed in this study was close to the value reported by Krstin et al. [55] for Croatian and Slovenian CHV1 populations. The nucleotide diversity was also similar to the values previously reported for CHV1 populations from Switzerland, Slovenia, Croatia, and North Macedonia [24,55]. Both partially sequenced regions, ORF-A and ORF-B, had similar values of haplotype and nucleotide diversity. However, the analyzed part of ORF-B had a slightly higher number of variable sites than ORF-A. This is in contrast with the results of Mlinarec et al. [56] that showed the replication-associated large ORF-B protein to be the most conserved protein domain within the CHV1 genome. Nevertheless, unlike this study, where we used partial ORF-A and ORF-B sequences, Mlinarec et al. used whole-genome sequences of CHV1 isolates of different subtypes, thus generating a more reliable assessment of genetic variability of the species. Phylogenetic analyses of CHV1 populations from Montenegro, with no clear clustering of populations and relatively low bootstrap values, suggest a closely related population with a relatively recent common ancestor.

On a broader geographical scale, we analyzed CHV1 populations in the context of surrounding and nearby countries from which the sequences of the same part of the genome are available—North Macedonia, Bosnia and Herzegovina, and Croatia. We confirmed the complex structure of CHV1 populations in central Europe with intermixing of populations from Croatia and Bosnia and Herzegovina [55], and the clear clustering of Macedonian populations into a separate clade [24,55]. Montenegro, however, did not segregate into a separate clade, but most of the sequences obtained in this study did group together and were placed more basally, somewhat closer to reference strain EP713 (French subtype) than the other sequences. Several sequences were placed within the Croatian and Bosnian group. Due to the geographical position of analyzed populations, it would be expected that the North Macedonian CHV1 sequences would have been more closely related to the ones from Montenegro than the ones from Croatia or Bosnia and Herzegovina, but surprisingly, we found that the Montenegrin sequences were more distantly related to the Macedonian ones. Bryner et al. [35] proposed the central European origin of CHV1 in Macedonia, which then spread into the neighboring Greece. The lack of close relation between Montenegrin and North Macedonian populations, the more basal position of the Montenegrin sequences, as well as obvious reticulation of haplonetwork noted in our study may suggest a possibility of two independent introduction events of CHV1 into Montenegro. Possible routes of entry are Bosnia and Herzegovina or Croatia, but the separate, more basal CHV1 haplotypes could have even originated in southern Italy. Neighboring Albania had very intensive trade relations with Italy, the biggest European chestnut producer [57]. Unfortunately, the sequences of the Albanian CHV1 isolates are not available [43], thus making this conclusion only hypothetical. Further comparison of CHV1 populations from Montenegro, Albania, and southern Italy would provide more definite answers to exact CHV1 spread routes in Europe, and especially the Balkan peninsula.

## 5. Conclusions

The presence of several vc types and the diversity of *C. parasitica* populations in Montenegro indicate its longer presence in the country than previously thought. Luckily, in all studied populations, natural biocontrol with CHV1 was observed. However, the potential for a further increase in *C. parasitica* vc-type diversity due to the presence of both mating types makes these chestnut populations vulnerable to the blight disease, making further monitoring of the spread and population dynamics of this dangerous pathogen a necessity.

## Figures and Tables

**Table 1:** *Cryphonectria hypovirus 1* (CHV1) sequences and their accession numbers used in this study.

Country	Sample (Accession Number) *	Sampling Year
Bosnia and Herzegovina (BiH)	Iv5 (JX970117), Iv7 (JX970119), Iv21 (JX970129), Iv31 (JX970133), Iv48 (JX970143), Ka6 (JX970080, Ka9 (JX970082), Ka14 (JX970087), Ka20 (JX970092), Ka49 (JX970113)	2008–2010 [35]
North Macedonia (MCD)	Ra1 (JX969931), Ra6 (JX969934), Ra25 (JX969942), Ra35 (JX969950), Ra69 (JX969979)	2008–2010 [35]
	kal_2A_1_F (MT799064), kal_4A_2_F (MT799065), kal_12A_1 (MT799066), kal_13B_2 (MT799067), kal_17A_rep (MT799068), kal_20B_2 (MT799069), kal_25A_rep (MT799070), kal_28A_2 (MT799071), kal_28B_3 (MT799072), kal_29A_3 (MT799073), kal_31A_rep (MT799074), kal_31B_3_R (MT799075), kal_35A_2 (MT799076), kal_35B_1 (MT799077), kal_38A_2 (MT799078), smo_1B_3 (MT799079), smo_18A_2_rep (MT799080), smo_20B_3 (MT799081), smo_31B_3A (MT799082), smo_32B_1 (MT799083), smo_41B_3 (MT799084)	2014 [24]
Croatia (CRO)	ks_01_A2 (MT799046), ks_01_B1 (MT799047), ks_03_A3 (MT799048), ks_04_A1 (MT799049), ks_07_B2 (MT799050), ks_11_A2 (MT799051), ks_15_C2 (MT799052), ks_18_A2 (MT799053), ks_18_C2 (MT799054), ks_20_B3 (MT799055), ks_20_C3 (MT799056), oz_04_C2 (MT799057), oz_06_C3 (MT799058), oz_08_B2 (MT799059), oz_10_B2 (MT799060), oz_14_B2 (MT799061), oz_16_A2 (MT799062), oz_18_B1 (MT799063)	2014 [24]
Montenegro (MNE)	BK-K_16-1 (ON180782, ON180803), BK-K_16-2 (ON180783, ON180804), BK-K_30-1 (ON180784, ON180805), BK-S_01-2 (ON180785, ON180806), BK-S_07-1 (ON180786, ON180807), BK-S_07-2 (ON180787, ON180808), BK-S_07-3 (ON180788, ON180809), BK-S_29-3 (ON180789, ON180810), LS-K_03-3 (ON180790, ON180811), LS-K_04b-1 (ON180791, ON180812), LS-K_09-2 (ON180792, ON180813), LS-O_04-3 (ON180793, ON180814), LS-O_05-1 (ON180794, ON180815), LS-O_05-3 (ON180795, ON180816), LS-O_06-1 (ON180796, ON180817), LS-O_06-2 (ON180797, ON180818), LS-O_07-2 (ON180798, ON180819), LS-O_09-2 (ON180799, ON180820), LS-O_09-3 (ON180800, ON180821), LS-O_14-1 (ON180801, ON180822), LS-O_14-2 (ON180802, ON180823)	2019–2020 (this research)

* For BiH, MCD, and CRO, the accession numbers given are for sequences of ORF-A, and for MNE for each sample, both ORF-A and ORF-B sequences are given, in that order.

**Table 2:** Vegetative compatibility types of *Cryphonectria parasitica* isolates from four populations in Montenegro.

POPULATION	Cankers Sampled	Cankers Isolated ^a^	Multiple Isolates/Different vc Types ^b^	N ^c^	EU-1 ^d^	EU-2 ^d^	EU-4 ^d^	EU-5 ^d^	EU-6 ^d^	EU-8 ^d^	EU-10 ^d^	EU-11 ^d^	EU-12 ^d^	EU-17 ^d^	EU-22 ^d^	Vc Types
Bay of Kotor—Kostanjica	30	14 (46.7%)	5/2	20		5 (25.0)	1 (5.0)			1 (5.0)			13 (65.0)			4
Bay of Kotor—Stoliv	31	19 (61.3%)	3/1	23		9 (39.1)				1 (4.3)			11 (47.8)	1 (4.3)	1 (4.3)	5
Lake Skadar—Koštanjica	21	10 (47.6%)	1/1	11	1 (9.1)			3 (27.3)	2 (18.2)			1 (9.1)	4 (36.4)			5
Lake Skadar—Ostros	21	17 (81.0%)	7/1	25				1 (4.0)	1 (4.0)		2 (8.0)	2 (8.0)	18 (72.0)	1 (4.0)		6
TOTAL	103	60 (58.3%)		79	1 (1.3)	14 (17.7)	1 (1.3)	4 (5.1)	3 (3.8)	2 (2.5)	2 (2.5)	3 (3.8)	46 (58.2)	2 (2.5)	1 (1.3)	

^a^ Number of cankers from which *C. parasitica* was successfully isolated, with percentages shown in parentheses; ^b^ number of cankers with multiple isolates/number of different vc types found among those multiple isolates; ^c^ total number of isolates in each population; ^d^ EU type determined according to [13]; percentage of each EU type is shown in parentheses.

**Table 3:** Polymorphic *vic* loci, maximum number of vegetative compatibility types, population diversity measures, and mating types of *Cryphonectria parasitica* isolates from four populations in Montenegro. Statistically significant differences at *p* < 0.05 are denoted by an asterisk (*).

			Population Diversity Measures		Mating Type			
POPULATION	N Polymorphic *vic* Loci	Max. N of vc Types ^a^	*H′* ^b^	*E* ^c^	MAT1-1	MAT1-2	χ^2^	*p*
Bay of Kotor—Kostanjica	3	8	0.93 (0.71–1.23)	0.63 (0.51–0.86)	11	9	0.2	0.654720846
Bay of Kotor—Stoliv	3	8	1.13 (0.94–1.37)	0.62 (0.58–0.83)	21	2	15.70	<0.0001 *
Lake Skadar—Koštanjica	5	32	1.47 (1.17–1.59)	0.87 (0.71–0.98)	5	6	0.09	0.763024601
Lake Skadar—Ostros	6	64	1.03 (0.93–1.47)	0.46 (0.42–0.73)	23	2	17.64	<0.0001 *

^a^ Maximum number of vc types assuming sexual recombination at all polymorphic *vic* loci in a population; ^b^ Shannon diversity index *H*′; ^c^ Buzas and Gibson’s evenness $E=\frac{e^{H’}}{S}$.

**Table 4:** *Cryphonectria hypovirus 1* (CHV1) prevalence in *Cryphonectria parasitica* isolates from four populations in Montenegro.

Population	N Isolates (N Cankers) ^a^	Positive Isolates (Positive Cankers) ^b^	Prevalence per Isolate (Prevalence per Canker) (%) ^c^	vc Types ^d^
Bay of Kotor—Kostanjica	20 (14)	3 (2)	15.0 (14.3)	EU-12
Bay of Kotor—Stoliv	23 (19)	5 (3)	21.7 (15.8)	EU-2, EU-12, EU-17
Lake Skadar—Koštanjica	11 (10)	3 (3)	27.3 (30.0)	EU-5, EU-6
Lake Skadar—Ostros	25 (17)	10 (6)	40.0 (35.3)	EU-12
TOTAL	79 (60)	21 (14)	26.6 (23.3)	

^a^ Total number of *C. parasitica* isolates and number of cankers from which *C. parasitica* was successfully isolated (in parentheses) in each population; ^b^ number of CHV1-positive isolates and cankers (in parentheses); ^c^ prevalence (%) of CHV1 in each population, calculated per number of isolates or per number of cankers (in parentheses); ^d^ vegetative compatibility types in which CHV1 was detected.

## Data Availability

All data obtained in this research are available upon request. The sequences obtained in this study are deposited in GenBank under the accession numbers ON180782-ON180823.
